# Prise en charge des ruptures traumatiques des corps caverneux au sein d'une population militaire

**DOI:** 10.11604/pamj.2014.18.260.4732

**Published:** 2014-07-29

**Authors:** Mohammed Alami, Abdellatif Janane, Mohamed Ghadouane, Ahmed Ameur, Mohamed Abbar

**Affiliations:** 1 Service d'Urologie Hôpital Militaire Universitaire Moulay Ismail, Meknes, Maroc; 2 Service d'Urologie Hôpital Militaire Universitaire Mohamed V, Rabat, Maroc

**Keywords:** Rupture, corps caverneux, traitement, évolution, militaire, Rupture, Corpus cavernosum, treatment, Evolution, military

## Abstract

Le but de ce travail rétrospectif était de préciser les résultats de notre prise en charge des traumatismes du corps caverneux au cours des huit dernières années. De Janvier 2000 à Février 2008, 32 malades ont été opérés pour une rupture traumatique des corps caverneux. Nous avons analysé: l’âge, le mécanisme, le délai de consultation, la présentation clinique, la technique opératoire, le siège de la rupture et les lésions associées, l’évolution immédiate et les suites lointaines. Avec un recul moyen de 42 mois (12 à 84 mois), les patients étaient jeunes (23 à 51 ans), le faux pas du coït était le mécanisme le plus fréquent (21 malades) 65,6% suivi la tentative de dissimuler une érection (18,7%) (06maldes). Nos malades avaient tous consultés dans les heures qui suivaient (délai d'hospitalisation < 19 heures). 02/32 malades, soit 6,25%, avaient des urèthrorragies en plus de la présentation typique (hématome, déviation de la verge). La rupture albuginéale était située à droite chez 50% des patients, à gauche dans 43,7% (14cas) et bilatérale chez deux patients. Le siège de la rupture était proximal dans 100% des cas (32hommes). L'incision élective était notre choix dans 93,75% des cas (30 malades).. Les suites lointaines étaient favorables, hormis un gène sexuel les 06 premiers mois chez 37,4% (12maldes). La simple suture albuginéale après évacuation de l'hématome par voie élective, en dehors des cas compliqués de lésions urétrales ou vus tardivement, semblait apporter les meilleurs résultats anatomiques et fonctionnels.

## Introduction

Déjà observés il y a plus de mille ans par Abul Kacem à Cordoue, les ruptures des corps caverneux ont été décrites pour la première fois en 1925 [1, 2]. Il s'agit d'une urgence urologique rare survenant le plus souvent chez l'adulte jeune. Dans les pays occidentaux la cause la plus fréquente était de loin le « faux pas du coït ». Après avoir évacué l'hématome conséquent, le traitement a toujours consisté en une suture de l'albuginée. Une bonne anamnèse, un bilan préopératoire et une bonne exploration per opératoire permettaient de ne pas méconnaître d’éventuelles lésions associées [3, 4]. Certaines complications lointaines, quoique peu fréquentes, méritaient d’être soulignées (la dysfonction érectile, les coudures de la verge, les plaques sous-albuginéales). Les modalités de la prise en charge diagnostique, thérapeutique ainsi que l’évolution ont été détaillées à travers une revue de la littérature.

## Méthodes

De Janvier 2000 à Février 2008, 32 malades ont été admis dans notre service, par le billais des urgences, pour une fracture des corps caverneux. Après un interrogatoire bref, l'examen clinique complété par une échographie permettait de poser le diagnostic et d'effectuer un bilan lésionnel précis (le siège de la rupture, les lésions associées). L'hématome sous cutané avec déviation de la verge était présent constamment chez 100% des malades. Nous avons étudié rétrospectivement l’âge, le côté lésé, l’étiologie et le mécanisme du traumatisme (le faux pas du coït, la masturbation, la tentative de dissimuler une érection, les jeux et les acrobaties sexuelles..).

Nous avons analysé le délai de consultation, la présence de signes urinaires après l'accident (hématurie ou urètrorragies) ainsi que les éventuelles anomalies biologiques à l'admission (trouble de la crase). Le bilan radiologique permettait de déceler des fractures du corps caverneux de petite taille, l'hématome associé bien visible sous forme d'une collection échogène, le siège proximale de la lésion et la recherche de lésions scrotales associées. Le traitement chirurgical consistait en une évacuation de l'hématome, une hémostase élective ainsi qu'une suture de l'albuginée. L'incision était élective dans 84,4% des cas. Le suivi était clinique (régression de l'hématome, disparition de la déviation, qualité de la rectitude et de l’érection).

## Résultats

32 traumatismes du corps caverneux ont été pris en charge, dans notre service, entre Janvier 2000 et Février 2008. L’âge moyen était de 38,7 ans (23-51ans). Le recul moyen est de 42 mois (12-84 mois). La rupture post traumatique du corps caverneux intéressait préférentiellement l'homme jeune (87,5%) et se situait aussi bien à droite (50%) qu’à gauche (43,74%). La fracture bilatérale était notée chez 2 patients (6,25%).

Le délai de consultation aux urgences était compris entre 3 heures et 19 heures. Le faux pas du coït était le mécanisme le plus fréquent (21 malades) 65,6% suivi la tentative de dissimuler une érection (18,7%) (06maldes); chez 5 malades (16,6%) l’étiologie était occultée. L’évaluation clinique, à l'admission, avait mis en évidence chez tous les patients une déviation de la verge avec un hématome sous cutané (aspect aubergine de la verge). Deux malades (6,25%) avaient une urètrorragie avec hématome en aile de papillon suggérant l'atteinte urétrale.

Le bilan biologique, à la recherche de dyscrasie, était normal chez tous les patients. L’échographie pénienne, examen morphologique de première intention, avait confirmé le siège proximal chez 100% de nos malades. La rupture albuginéale était située à droite chez 50% des patients, à gauche dans 43,7% (14cas) et bilatérale chez deux patients. L'exploration chirurgicale immédiate sous rachianesthésie, par voie élective chez 30patients (93,75%), avait permis d’évacuer l'hématome et de faire l'hémostase des vaisseaux qui saignent. La réparation de l'albuginée était ensuite faite par du fil résorbab1e 3/0.

Chez deux de nos malades la suture albuginéale bilatérale était associée à une suture de la lésion spongieuse sur sonde urétrale charrière 18; celle-ci a été retirée au 7ème jour. Hormis ces deux cas, le séjour hospitalier moyen était court (inférieur à 3 jours). La prescription de benzodiazépines, destinés à limiter les érections, pendant le séjour hospitalier et la période de convalescence à domicile était systématique. Concernant l’évolution: les suites lointaines étaient favorables; hormis un gène ou un inconfort sexuel, rapporté durant les 06 premiers mois, chez 37,4% (12maldes). Aucun cas d'infection, ou d'abcédation n'a été répertorié. 100% des patients étaient satisfaits de la qualité de leur érection et rapport sexuelle.

## Discussion

Le traumatisme des corps caverneux du pénis fut décrit la première fois en 1925. Cette affection peu fréquente est, de loin, principalement observée chez l'adulte jeune; avec une moyenne d’âge de 30 ans et des extrêmes allant de (11 à 83 ans). ISHIKA W. A rapportait dans sa série que 91% des malades avaient moins de 50 ans: âge où les rapports sexuels sont plus fréquent en quantité et plus vigoureux en qualité [5, 6]. A titre anecdotique, 73% des hommes n’étaient pas mariés dans la série d'EKE [7].

La plus grande série rapportée dans la littérature concerne la série saoudienne d'AGRA avec 292 cas [2]. Pour certains auteurs, l'incidence serait en nette augmentation depuis l'apparition de traitement efficace de l'impuissance sexuelle (le sildénafil): ils avaient compté en moyenne un cas par semaine aux urgences du centre hospitalier universitaire [8, 9].

Quant aux étiologies: à cause de la gêne du patient, les circonstances exactes de la survenue étaient parfois difficiles à faire préciser. En occident et en Amérique latine, la cause la plus fréquente était un rapport sexuel vaginal vigoureux: le pénis en érection vient percuter la symphyse pubienne de la partenaire lorsqu'il glisse hors du vagin; le coït en position verticale pouvait aussi entraîner une fracture du pénis lorsque la partenaire tombait soudainement. Enfin la lésion a été décrite lors de jeux et d'acrobaties sexuelles [10, 11]. Au Maghreb et au Moyen Orient, c’étaient surtout les manipulations visant à stopper l’érection matinale, ainsi que la masturbation, qui ont été cité comme les accidents les plus fréquents [12].

La physiopathologie ou la biomécanique en cause était surtout la courbure brusque tonique, non physiologique, s'exerçant sur l'axe du pénis et entraînant une déchirure de l'albuginée des corps caverneux. Cette dernière est amincie et plus vulnérable en phase de rectitude [13, 14]. Certaines ruptures ont été étendues au corps spongieux et à l'urètre.

La topographie du trait de fracture correspond était le plus souvent unilatéral et transversal suivant la disposition des fibres de collagènes et non celle des fibres d’élastine; sans que cela puisse être expliqué [9, 11]. Le siège était proximale- dorsale dans la plupart du temps (87%) et plus rarement au niveau du tiers distal. La bilatéralité était exceptionnelle s'accompagnant à fortiori d'une lésion urétrale concomitante. La rupture partielle ou totale de l'urètre était présente dans 1,3 à 2,8% des cas. [15] Un trait de fracture de l'albuginée du corps caverneux, de 10 à 25 mm, pouvait générer un hématome étendu du périnée à la région sus-pubienne. La rupture urétrale synchrone devait être recherchée: lors d'une fracture bilatérale, d'une violence du faux pas du coït et surtout lorsque cette dernière s'accompagne d'une urètrorragie [16].

La première étape diagnostique était l'interrogatoire qui devait préciser les circonstances exactes du traumatisme. Le délai moyen de consultation, comme dans notre série, était généralement inférieur à 24 heures. Sur un pénis en érection, la fracture générait un bruit de craquement soudain évoquant la rupture d'une tige de maïs; ensuite s'installait une détumescence rapide et un hématome sous cutané progressif: « pénis en aubergine» [16, 17].

A l'examen clinique, une déviation de l'axe du pénis du côté opposé à la fracture et une dépression palpable à travers les enveloppes au niveau du site de fracture. Le « Rolling Sign » était un indice qui permettait d'identifier le site de fracture: le caillot qui se formait au niveau de la fracture était palpable sous la peau du pénis qui se roulait dessus [18, 19]. Un hématome périnéal en aile de papillon avec une urètrorragie ou des brûlures mictionnelles signait l'atteinte urétrale.

Vue tardivement, la maladie était devenue surtout une dysfonction érectile avec coudure de la verge associée ou non à des plaques; elle prêtait à confusion avec la maladie de La Pyeronie. D'autres complications, de la fracture de la verge vu à distance de l'accident, avaient été rapportés dans la littérature: la fistule rétro-caverneuse, la fistule urètro-cutanée ou encore la sténose de l'urètre.

Le diagnostic de la rupture du pénis reposait souvent sur l'anamnèse et l'examen physique. La place controversée de l’échographie (sonde superficielle de haute fréquence 7,5-MHz), accessible au niveau des urgences, pouvait préciser le siège de la rupture et sa direction. Elle mettait en évidence le caractère échogène de l'hématome pure; ainsi que son étendu intra ou extra caverneux [17, 18].

L'imagerie par résonance magnétique avait donné des résultats prometteurs dans cette pathologie traumatique. Cet examen déjà exploité dans le bilan d'extension locale des tumeurs du pénis ainsi que dans la maladie de La Pyeronie [19, 20]. Cependant, dans notre situation nous ne l'avons jamais pratiqué: il était considéré comme un examen coûteux, de disponibilité réduite et qui pouvait allonger le délai de la prise en charge chirurgicale de nos patients. Les auteurs l'ayant réalisé maintenaient le pénis dans un support ce qui permettait de l'observer dans une position proche de l’érection, sans recourir à une érection artificielle en phase aiguë [20]. Cet examen nucléaire offrait une évaluation correcte des deux segments (proximale et distale): la solution de continuité était visible sous la forme d'un hyposignal en Tl, non détectable en T2. L'hématome sous cutané était aussi bien décelable en Tl qu'en T2; en revanche l'hématome intra-caverneux était mieux analysé en séquence pondérée Tl [20, 21]. Pendant cette même séquence, la muqueuse de l'urètre devait renvoyer un hypersignal précoce qui permettait de suivre son trajet et de mettre en évidence le moindre défect muqueux. L'IRM a été utilisée dans le suivi de cette affection traumatique pour confirmer la bonne cicatrisation des tissus lésés et réparés.

ZARGOOCHI [20], avait proposé une urètrocystographie, avant chirurgie, aux malades présentant une urètrorragie; les limites de cet examen étaient: Les faux négatifs en raison de la compression urétrale par l'hématome caverneux; Elle pouvait transformer une lésion urétrale partielle en rupture totale; L'absence d'extravasation, en cas de contexte clinique évocateur, n’éliminait pas la rupture; ce qui justifiait un contrôle endoscopique peropératoire.

En 1936, FETTER fut le premier à rapporter le premier cas de traitement chirurgical de rupture du corps caverneux. Plus tard, TAHA défendra les avantages d'une telle prise en charge [19]. A l'heure actuelle, le traitement de référence de cette entité urogénitale est basé sur l’évacuation de l'hématome associée à la suture de la déchirure albuginéale.

Sous rachianesthésie ou anesthésie générale, plusieurs voies d'abords ont été décrites [20, 21]: (1)L'incision coronale au niveau du sillon balano-préputial avec dégantage complet du pénis, offrait l'avantage d'un large accès exposant les deux corps caverneux et le corps spongieux, comportait le risque infectieux et œdémateux ainsi que la nécrose cutanée (11 à 20%); (2)L'incision élective proximale, évitait la lésion des rameaux nerveux sensitifs balano-préputials, la cicatrice était inéluctablement inesthétique; (3)L'incision scrotale haute, plus esthétique et autorisant le dégantage pénien, pouvait être utilisée pour les fractures proximales.

Les principes du traitement chirurgical étaient: l’évacuation de l'hématome, l'hémostase et le parage-suture de l'albuginée du corps caverneux. Pour le saignement d'origine veineux, la ligature était souvent préférée à la coagulation électrique (risque de nécrose urétrale). En cas de plaie artérielle, la ligature était choisie au lieu de l'artérialisation de la veine dorsale du pénis; sa réalisation ne permettait pas d’éviter la dysfonction érectile. L'impuissance, après cure de rupture de verge, semblait être en rapport avec une fuite veineuse vers le corps spongieux: ceci rendait alors illusoire les tentatives de réparation artérielle [21, 22].

La suture de l'albuginée se faisait généralement en suivant l'axe longitudinal du pénis à l'aide de points séparés de fil résorbable 3/0 ou 4/0. Le fil non résorbable, réservé au rares cas de récidive dans la série de Zargoochi, utilisé en points séparés inversant pour éviter l'inconfort de les palper en sous cutané [20]. La cicatrisation était obtenue au bout de 06 semaines, des phénomènes de réorganisation architecturelle se poursuivaient pendant deux ans. En cas de lésion caverneuse bilatérale et ou urètrorragie, une urètroscopie per-opératoire est recommandée suivie d'une éventuelle suture urétrale sur sonde tutrice; cette dernière sera gardée en moins pendant 08 jours [18]. Le pansement devait laisser visible le gland pour identifier précocement un problème ischémique. La prescription post-opératoire quotidienne de diethystilbestrol, visant à éviter les érections source de lâchage de suture, était recommandée pendant 2 semaines [21, 22].

Le traitement chirurgical était unanimement la procédure de référence; mais il n'en était pas moins vrai que le traitement médical (antalgiques, anti-œdémateux, anti-androgènes, benzodiazépines et compresses froides) pouvait être proposé dans certaines situations exceptionnelles: Contexte évocateur de rupture caverneuse avec présentation clinique non classique plutôt fruste (absence d'hématome et de déformation); Refus total par le patient de subir un geste chirurgical.

Le taux de complications dans le traitement conservateur (médical) variait de 11 à 34%: surtout des coudures douloureuses au moment de l’érection. L'impuissance pouvait être imminente dans certains cas (24 à 47%). D'autres suites malencontreuses, quoi que peu fréquentes, méritaient d’être soulignées: l'hématome infecté ou abcédé, la fistule artério-veineuse et la sténose urétrale d'origine compressive (l'hématome infecté non résorbé) [22].

## Conclusion

La rupture de l'albuginée des corps caverneux était une urgence urologique peu fréquente; elle survenait souvent chez l'adulte jeune. Le tableau clinique ne se faisait aucun doute, l’échographie permettait un bilan lésionnel précis (le siège exact du trait de fracture), l'IRM pouvait être proposé pour le suivi permettant d'affirmer ou d'infirmer une bonne cicatrisation. Les principes du traitement étaient: l’évacuation de l'hématome sous cutané, la suture de l'albuginée de préférence par une incision élective. Le pronostic de la maladie dépendait toujours de l'existence ou non de lésion urétrale, lésion artérielle ou veineuse majeure. Vue tardivement, elle exposait au risque de fistule caverno-spongieuse, de sténose urétrale due à l'hématome compressif, de dysfonction érectile et de coudure de verge.
